# Computational Approaches to Understanding the Role of Fibroblast-Myocyte Interactions in Cardiac Arrhythmogenesis

**DOI:** 10.1155/2015/465714

**Published:** 2015-10-25

**Authors:** Tashalee R. Brown, Trine Krogh-Madsen, David J. Christini

**Affiliations:** ^1^Department of Physiology, Biophysics, and Systems Biology, Weill Cornell Medical College, New York, NY 10065, USA; ^2^Weill Cornell/Rockefeller/Sloan-Kettering Tri-Institutional MD-PhD Program, New York, NY 10065, USA; ^3^Greenberg Division of Cardiology, Weill Cornell Medical College, New York, NY 10065, USA

## Abstract

The adult heart is composed of a dense network of cardiomyocytes surrounded by nonmyocytes, the most abundant of which are cardiac fibroblasts. Several cardiac diseases, such as myocardial infarction or dilated cardiomyopathy, are associated with an increased density of fibroblasts, that is, fibrosis. Fibroblasts play a significant role in the development of electrical and mechanical dysfunction of the heart; however the underlying mechanisms are only partially understood. One widely studied mechanism suggests that fibroblasts produce excess extracellular matrix, resulting in collagenous septa. These collagenous septa slow propagation, cause zig-zag conduction paths, and decouple cardiomyocytes resulting in a substrate for arrhythmia. Another emerging mechanism suggests that fibroblasts promote arrhythmogenesis through direct electrical interactions with cardiomyocytes via gap junctions. Due to the challenges of investigating fibroblast-myocyte coupling in native cardiac tissue, computational modeling and *in vitro* experiments have facilitated the investigation into the mechanisms underlying fibroblast-mediated changes in cardiomyocyte action potential morphology, conduction velocity, spontaneous excitability, and vulnerability to reentry. In this paper, we summarize the major findings of the existing computational studies investigating the implications of fibroblast-myocyte interactions in the normal and diseased heart. We then present investigations from our group into the potential role of voltage-dependent gap junctions in fibroblast-myocyte interactions.

## 1. Introduction

One of the hallmarks of aging and heart disease is the structural remodeling of the heart by an increased density of cardiac fibroblasts (fibrosis). Fibroblasts are flat, spindle-shaped cells with long processes that form a network of cells surrounding cardiomyocytes. Fibroblasts are a phenotypically heterogeneous population of cells [1] and their phenotype varies in response to the pathological conditions of the heart. Only activated fibroblasts, termed myofibroblasts, express *α*-smooth muscle actin and display levels of contractility, proliferation, and collagen synthesis that are enhanced compared to nonactivated fibroblast. This differentiation is promoted by cytokines such as TGF-*β*1 which are released in response to cardiac injury and mechanical stress and can be induced experimentally by certain cell culture conditions [2].

Fibroblasts play a significant role in the development of electrical dysfunction of the heart during disease states such as myocardial infarction and various cardiomyopathies; however the mechanisms are only partially understood. One widely studied mechanism suggests that fibroblasts produce excess extracellular matrix, resulting in collagenous septa. These collagenous septa slow propagation, cause zig-zag conduction paths, and decouple cardiomyocytes resulting in a substrate for arrhythmogenic activity [3]. Another emerging and somewhat controversial mechanism suggests that fibroblasts promote arrhythmogenesis through direct electrical interactions with cardiomyocytes via gap junction (GJ) channels.

Several* in vitro* experiments using normal and diseased heart models have demonstrated that fibroblasts make direct electrical interactions with cardiomyocytes via GJ channels [4–7]. For example, using a dye transfer assay, Baudino et al. [7] showed in a three-dimensional cell culture model of neonatal rat cardiomyocytes and fibroblasts that cell-cell interactions exist between fibroblasts and cardiomyocytes. Furthermore, Vasquez et al. [6] used a gap fluorescence recovery after photobleaching technique to show that intercellular coupling was enhanced between cardiomyocyte monolayers cocultured with cardiac fibroblasts derived from infarcted rat hearts compared to cardiac fibroblasts derived from normal hearts.

However, a major challenge in the field has been to translate such cell culture discoveries into native cardiac tissue and the whole heart. Camelliti et al. used immunolabeling and a scrape-loading dye transfer method to demonstrate that fibroblasts and cardiomyocytes are functionally coupled in the rabbit sinoatrial node [8]. However, Baum et al., using a similar method, found no fibroblast-myocyte (F-M) coupling in a canine model of myocardial infarction [9]. To date, it is unsettled whether F-M coupling exists* in vivo* and whether such discrepancies are due to regional differences (sinoatrial node versus ventricle), species related differences (rabbit versus canine), or disease related modifications in F-M coupling (see [10, 11] for two recent reviews on this topic).

The intermingled structure of cardiomyocytes and fibroblasts in native cardiac tissue has made it difficult to study their interactions* in vivo*; thus computational modeling and* in vitro* experimental approaches have been the main method used to investigate the arrhythmogenic implications of their potential interactions. In this review, we summarize the characteristics and major findings of the existing multiscale computational models of F-M interactions in normal and diseased heart models and highlight their utility in providing mechanistic insights into experimental investigations.

In the next section, we review the existing computational models of the electrophysiological properties of ventricular and atrial fibroblasts and discuss some of the experimental basis of their development.

## 2. Mathematical Models of Cardiac Fibroblasts

A major advancement in our understanding of F-M interactions was the discovery that cardiac fibroblasts express time- and voltage-dependent and inward rectifying K^+^ currents [12, 13]. Computational models incorporating the electrophysiological properties of these conductances are described as “active” models. Prior to this discovery, cardiac fibroblasts were modeled as purely “passive” electrical loads.

### 2.1. Ventricular Fibroblast Model

#### 2.1.1. Passive Model

In the passive fibroblast model, the membrane capacitance is connected in parallel to an ohmic resistance. Therefore, the membrane potential can be represented by the ordinary differential equation: *C*_f_(*dV*_f_/*dt*) = −*G*_f_(*V*_f_ − *E*_f_), where *C*_f_ is the fibroblast membrane capacitance, *V*_f_ is the membrane potential of the fibroblast, *E*_f_ is the fibroblast resting membrane potential, and *G*_f_ is the fibroblast membrane conductance [14]. This model does not accurately represent all of the electrophysiological properties of cardiac fibroblasts. However, due to the ability to independently modify *E*_f_ and *G*_f_, this model has been extensively used to systematically investigate these basic fibroblast electrophysiological parameters in multiscale computational models of F-M interactions (see Section 3.1.1).

#### 2.1.2. Active Model

Active fibroblast models have been developed by Sachse et al. [15], Jacquemet and Henriquez [16], and MacCannell et al. [17], with the MacCannell model being the most widely used. The MacCannell et al. model includes four membrane currents: an inwardly rectifying K^+^ current, a time- and voltage-dependent delayed-rectifier K^+^ current, an electrogenic Na^+^/K^+^-ATPase, and a time-independent background Na^+^ conductance. The magnitude and kinetics of the inwardly rectifying and delayed-rectifier K^+^ currents are based on experimental measurements [12, 13] from fibroblasts isolated from the adult rat ventricle. The Na^+^/K^+^-ATPase and the background Na^+^ current were introduced to enable K^+^ and Na^+^ ion homeostasis. The resting membrane potential of the uncoupled fibroblast is set to −49.6 mV and the membrane capacitance is 6.3 pF.

The Sachse model is based on the same experimental data as the MacCannell model and therefore also includes an inwardly rectifying current *I*_Kir_ and a time- and voltage-dependent outward current *I*_Shkr_ (i.e., the *K*_*v*_ family) but with different mathematical formulations. They also incorporated a nonspecific background current *I*_*b*_ to maintain the resting membrane potential of −58 mV and they modeled a smaller membrane capacitance of 4.5 pF. The Jacquemet model is a simplified active model developed by fitting a three-dimensional polynomial to the recorded current-voltage relationship of the cardiac fibroblast and incorporating a delayed current activation. The resting membrane potential was set to −58 mV.

### 2.2. Atrial Fibroblast Model

Recent experimental studies implicate potentially important differences between cardiac fibroblasts derived from ventricular versus atrial tissue [18–20]. This has led to a subset of models representing the atrial fibroblast phenotype. Chatelier et al. have shown that the differentiation of human atrial fibroblasts into myofibroblasts is associated with* de novo* expression of voltage gated Na^+^ currents [19, 20]. Koivumäki et al. [20] integrated these electrophysiological findings into a mathematical model of human atrial myofibroblasts [21]. However, the introduction of Na^+^ currents into the model did not result in significant changes in the fibroblast electrophysiology nor any action potential-like responses on stimulation. This may be due to the inactivation of the Na^+^ current by the relatively depolarized resting membrane potential of the cardiac fibroblast.

There are still significant gaps in our understanding of fibroblast electrophysiological properties due to the limited availability of electrophysiological data and the difficulty of accurately recording from such small cells [22]. In an attempt to overcome such limitations, computational studies have explored the contribution of a wide range of fibroblast properties by varying parameters such as membrane capacitance, *C*_f_, membrane conductance, *G*_f_, resting membrane potential, *E*_f_, gap junctional conductance, *G*_j_, and F-M ratio and quantifying their effects on F-M coupling.

## 3. Simulations of Fibroblast-Myocyte Coupling

Cardiac fibroblasts have been described as a “leaky capacitor”: charging during the diastolic and upstroke phase of the action potential (AP) and then leaking current during the systolic phase when the voltage-dependent currents of the cardiac fibroblast are activated by depolarization [23]. This in turn can cause modifications in the cardiomyocyte AP morphology when/if coupled to a fibroblast. Three hypothetical types of F-M coupling configurations in the intact heart have been proposed [24]. First, “zero-sided” coupling in which fibroblasts do not interact directly with cardiomyocytes but instead create obstacles similar to collagenous septa leading to discontinuous conduction. Second, “single-sided” coupling in which fibroblasts are connected to groups of myocytes and can act as current sources or sinks. Third, “double-sided coupling” in which fibroblast-fibroblast connections interlink myocytes resulting in new conduction pathways or conduction bridges between uncoupled myocytes. In this section, we review the existing computational models of F-M coupling in the form of cell pairs/clusters (i.e., a single cardiomyocyte coupled to one or more cardiac fibroblasts), one-dimensional (1D) cables, two-dimensional (2D) sheets, and three-dimensional (3D) models, and discuss their contribution to our understanding of the mechanism of F-M interactions.

### 3.1. Ventricular Tissue Models

#### 3.1.1. Effects on AP Morphology

MacCannell et al. [17] used computational models of cell pairs using the Tusscher-Noble-Noble-Panfilov (TNNP) model of the human ventricular cardiomyocyte [25] and their active model of an adult ventricular fibroblast (Section 2.1) to show that F-M coupling modifies the cardiomyocyte AP morphology. F-M coupling resulted in a hyperpolarized AP plateau, shortened AP duration (APD), depolarizations of the resting membrane potential, and corresponding AP waveform-dependent changes in the ionic currents of the cardiomyocyte. They found that the magnitude of these changes in AP was dependent on the membrane properties of the cardiac fibroblasts, the gap junction conductance, and the number of fibroblasts. The dependency of these AP changes on fibroblast membrane properties were further explored by Xie et al. [26] who described such changes as a function of the two components of the F-M gap junctional current: (1) an early transient outward (*I*_to_)-like component and (2) a late background current component. They performed simulations using a modified version of the Luo and Rudy (LR1) model [27] and the passive fibroblast model (Section 2.1.1) and systematically modified the fibroblast membrane conductance, *G*_f_, and resting membrane potential, *E*_f_, and observed its effects on the two components of the gap junctional current and on AP morphology during F-M coupling. They found that when *G*_f_ is small, the early component of the the gap junctional current behaves similar to *I*_to_ (i.e., it is activated rapidly during the early phase 1 of the AP and can influence AP amplitude and APD) and results in prolongation of the APD. When *G*_f_ is large, the late component of the gap junctional current plays a more prominent role in modifying the APD. The parameter *E*_f_ affects the crossing voltage in which the fibroblast voltage is more depolarized than the cardiomyocyte membrane potential. Therefore, when *G*_f_ is large and *E*_f_ is more depolarized, the late component of the gap junctional current is mainly an inward current for the cardiomyocyte resulting in the prolongation of its APD. However, when *G*_f_ is large and *E*_f_ is more hyperpolarized (i.e., −80 mV) the late component is mainly an outward current and thus shortens APD.

As will be discussed in the next section, F-M coupling can also have significant effects on cardiac impulse propagations and cardiomyocyte excitability as demonstrated by* in vitro* experiments and simulations using 1D cables and 2D tissue sheets.

#### 3.1.2. Effects on CV and Excitability

*CV and Fibroblast Density*. Experimental investigations* in vitro* have demonstrated that myofibroblasts can directly modify conduction velocity (CV) by direct electrical interactions with cardiomyocytes. For example, Miragoli et al. [28] measured CV and upstroke velocity (*dV*/*dt*_max_) from optical mapping recordings of stimulated strands of cultured neonatal rat cardiomyocytes coated with myofibroblasts. Interestingly, they found a biphasic dependence of CV and *dV*/*dt*_max_ on myofibroblast density. Using microelectrode recordings, they demonstrated that these fibroblast-mediated changes were associated with cardiomyocytes strands becoming depolarized from −78 mV to −50 mV.

To help explain these findings, Xie et al. [23] developed various 2D tissue sheet models based on native cardiac tissue structure in order to investigate the effects of F-M ratio on CV. In their cell-attached model, in which a layer of cardiac fibroblasts were modeled on top of a monolayer of cardiomyocytes, they observed a similar biphasic relationship between CV and F-M ratio as seen in* in vitro* experiments. They suggested that CV first increased by the fibroblast bringing the cardiomyocyte membrane potential closer to the threshold for *I*_Na_ but then decreased as the increasing fibroblast density resulted in a shift in the cardiomyocyte membrane potential and *I*_Na_ inactivation. However, using their random fibroblast insertion model, which represents the coculture of cardiomyocytes interspersed with cardiac fibroblasts, they found a monotonic decrease in CV with increasing F-M ratio. They also found that the membrane potential of the fibroblast, *E*_f_, also has an effect on CV: when *E*_f_ was set to a more hyperpolarized value of −80 mV the fibroblast had no effect on the cardiomyocyte resting membrane potential and there was a more linear relationship between CV and the F-M ratio.

However, these results were the opposite of the experimental findings of Miragoli et al., who found a biphasic relationship with endogenous/interspersed F-M cocultures, but a monotonic relationship when cardiac fibroblasts were plated on top of a cardiomyocyte monolayer. One suggested explanation of this discrepancy is that in experimental conditions where cardiac fibroblasts were plated on top of a cardiomyocyte monolayer, the latter may have still had significant endogenous/interspersed fibroblast content which might be high enough to obscure the increasing CV phase. Other yet to be explored mechanisms may also exist.

It has been shown that cardiac tissue microstructure consists of laminar clefts or cleavage planes with fibroblasts often localize in the cleft spaces. Surprisingly, their 2D model of fibroblasts inserted into laminar clefts between cardiomyocytes resulted in a monotonic increase in CV with increasing fibroblast density [23]. This behavior is thought to be due to cardiac fibroblasts forming bridge-like pathways or due to the downstream depolarization of a cardiomyocyte by a depolarized cardiac fibroblast within the cleft.

In order to further explore how fibroblasts may contribute to cardiac electrophysiology, Sachse et al. [29] extended the bidomain by describing both myocytes and fibroblasts and the surrounding extracellular space. This model allowed them to explore how fibroblasts affect extracellular potentials and to represent the spatial substitution of myocytes by fibroblasts during cardiac fibrosis. Interestingly, their simulations of conduction in a thin tissue slice showed that interfibroblast coupling had a greater effect on transverse than longitudinal CV. High fibroblast-fibroblast coupling resulted in a higher transverse CV compared to no fibroblast-fibroblast coupling, which indicates that the fibroblast domain can contribute to conduction. However, due to the lack of experimentally measured values, several modeling parameters were estimated or were varied over a broad range such as the volume fraction of myocytes, fibroblast, and extracellular space and the intrafibroblast conductivities.

*CV and Gap Junctional Conductance*. Zlochiver et al. [30] used a combination of* in vitro* optical mapping experiments and computational modeling studies to investigate the effects of F-M gap junctional conductance on CV. Gap junctional conductance was modified experimentally using gene expression level modification of connexin 43 (Cx43) channels, in which, Cx43 RNA interference resulted in a 90% reduction in myofibroblast Cx43 expression, and Cx43 overexpression resulted in 99% overexpression of Cx43. Their 2D computational model, designed to mimic the cell cultures experiments, used a ventricular cardiomyocyte model and the cardiac fibroblast model that included the outward rectifying current-voltage relationship (Section 2.1.2). In both simulations and experiments, there was a biphasic relationship between CV and gap junctional conductance, with an initial decrease in CV followed by an increase as gap junctional conductance increased. One hypothesis for the biphasic nature of this relationship is that insufficient charge is being transmitted to downstream cardiomyocytes during low levels of coupling, resulting in alternative conduction pathways and conduction slowing. However, above a certain threshold of gap junction conductance there is enough charge transmitted through the myofibroblasts to excite downstream cardiomyocytes and thus increase CV.

*Spontaneous Activity*. Miragoli et al. [31] used strands of cultured neonatal rat ventricular cardiomyocytes coated with myofibroblasts to demonstrate that F-M coupling can result in depolarization-induced ectopic activity. The percentage of preparations with spontaneous activity increased with increasing myofibroblast density. Such activity was not observed in control cardiomyocyte strands without direct myofibroblast contact. They demonstrated that changes in membrane polarization can affect the rate of occurrence of spontaneous activity by using current clamp injections of constant current pulses in individual cardiac myocytes in the range of membrane depolarizations seen in fibroblast coated cardiomyocyte cultures.

Greisas and Zlochiver [32] used a 2D monolayer tissue model, including human ventricular cardiomyocytes represented by the TNNP model [25] and the MacCannell cardiac fibroblast model and added support to the hypothesis that spontaneous activity occurs as a result of fibroblast-mediated depolarization of the cardiomyocyte resting membrane potential. In their monolayer tissue model, cardiac fibroblasts were embedded between ventricular cardiomyocytes in a single layer. In this configuration, spontaneous excitations were observed frequently. For example, spontaneous activity occurred with low F-M ratios from 0.5 to 1.75 with gap junctional conductances of 0.02 nS to 0.08 nS. The spontaneous excitation occurred in clusters when the coupled fibroblast modified the membrane potential of the neighboring cardiomyocytes to their excitation threshold. At low coupling the fibroblast effect was not strong enough to depolarize the cardiomyocyte, while at very high coupling the large depolarization and inactivation of sodium channel activity prevented the cardiomyocytes from recovering from inactivation after the AP was elicited. Similarly, automaticity was suppressed by very high fibroblast density due to the inability of the cardiomyocytes to recover from the large depolarization imposed by the coupled cardiac fibroblasts. Thus, the spontaneous activity only occurred at intermediate values of gap junctional conductance and when the fibroblast density was low.

#### 3.1.3. Effects on Vulnerability to Reentry

Majumder et al. [33] developed a computational model of 2D tissue of human ventricular cardiomyocytes using the TNNP model with randomly inserted passive cardiac fibroblast models. They investigated the effects of F-M coupling on spiral-wave dynamics and found multiple dynamical states as a function of initial conditions, boundary effects, and fibroblast density.

Heart failure (HF) can arise from numerous cardiac pathologies and can result in various degrees of electrical and structural remodeling depending on the particular etiology. Ionic remodeling and fibrosis have been identified as key players in the mechanisms for arrhythmogenesis associated with HF. Gomez et al. modeled ionic and structural remodeling in HF using 1D cables and 2D computational models [34, 35]. They used the Grandi et al. [36] and the O'Hara et al. [37] human ventricular AP model with HF ionic remodeling and the MacCannell fibroblast model for structural remodeling. When clusters of fibroblasts were inserted randomly into a cardiomyocyte strand APD dispersion increased to 70 ms from 24 ms with 10% fibrosis. Such regional dispersion of repolarization can create a substrate for the development of reentry. Moreover, transmural dispersion of repolarization (TDR) was also enhanced. Both APD dispersion and TDR showed a biphasic relationship with fibroblast density, with both APD dispersion and TDR first increasing with 10% fibrosis but then decreasing when fibroblast density was increased to 20%.

Furthermore, they investigated the role of fibrosis in reentry generation in several degrees of fibrosis [35]. They found that spontaneous activity occurred as a result of the depolarization of the cardiomyocyte membrane potential by the surrounding fibroblasts. In their HF remodeling simulations they found that low fibrosis (4%) could not induce reentrant activity, but at 14.5% there was a vulnerable window for reentry initiation of 20 ms. Thus, when enough fibrosis is present, the APD and effective refractory period of some cardiomyocytes are shortened so that when a premature stimulus is applied, part of the ventricular tissue has recovered enough to become excited, generating wave break and spiral waves. However, at even higher fibrosis (40%) reentrant activity was not observed because the depolarizing wave front reached very low potentials, leading to only small electronic voltage changes. They also observed a slight decrease in the rotation frequency of the spiral wave with increased fibrosis. This decrease in spiral wave frequency was also observed in experimental studies by Zlochiver et al. [30] and is consistent with fibroblast-loading induced conduction slowing (Section 3.1.2).

*Realistic 3D Computational Models*. Using a 3D computational model based on diffusion tensor magnetic resonance images of the rabbit heart, McDowell et al. [38] investigated the role of F-M coupling in the mechanism of arrhythmia generation during myocardial infarction. The peri-infarct zone was modeled as having a fibroblast density ranging from 10–30%, while the scar was model as 80% or 0% fibroblast density. Using the Mahajan et al. [39] model of the rabbit ventricular AP and the MacCannell fibroblast model, they showed that susceptibility to arrhythmia in the infarcted heart depends on myofibroblast density. At low densities myofibroblasts did not alter arrhythmia propensity, at intermediate densities myofibroblasts caused additional APD shortening and increased arrhythmia propensity, at high densities myofibroblasts protected against arrhythmia by causing resting depolarization and blocking propagation. The underlying mechanism was shown to be F-M coupling results in depolarization of the resting membrane potential of the cardiomyocyte, which causes a partial inactivation of *I*_Na_ and contributes to conduction failure.

#### 3.1.4. Effects on EADs and Cardiac Alternans

Since it is difficult to modify gap junctional conductance systematically in an* in vitro* experiment, Nguyen et al. [40] used a hybrid computational modeling and dynamic patch-clamp approach to investigate F-M coupling between a real rabbit ventricular cardiomyocyte and a virtual cardiac fibroblast. They showed that F-M coupling increased susceptibility to both oxidative stress-induced and hypokalemia-induced early afterdepolarizations (EADs) [40]. They deduce that these effects where dependent on the early *I*_to_-like component of the gap junctional current by performing experiments in which they selectively eliminated the early component and observed suppression of EADs. The *I*_to_-like component results in lowering of the AP plateau into the range that allows for reactivation of L-type calcium current. They also found that the increased EAD susceptibility was especially enhanced when the resting membrane potential of the cardiac fibroblast, *E*_f_, was more depolarized (−25 mV). It is worth noting that much smaller changes in F-M coupling were needed to result in EAD formation compared to effects on CV.

Xie et al. [26] used a computational model of F-M pairs and a 2D tissue modeled with random fibroblast insertions representing diffuse fibrosis to investigate the effects of F-M coupling on cardiac alternans, which have been linked to cardiac arrhythmogenesis. Using the LR1 model, they found that depending on the relative magnitude of the early and late components of the gap junctional current, F-M coupling can promote or suppress voltage-driven alternans and provide a novel mechanism for APD alternans at slow heart rates. The gap junctional current has been shown to be similar to the fast *I*_to_, and thus the mechanism of alternans is thought to be similar. This mechanism of alternans involves an interplay between the L-type calcium current and *I*_to_ current [41].

Furthermore, they showed with the Mahajan model [39] that F-M coupling can also promote calcium-driven alternans and spatially discordant alternans [26]. They found that spatially discordant alternans can occur by two mechanisms during F-M coupling, due to spatial variations in CV and due to spatial heterogeneity in fibroblast density resulting in regions that are electromechanical concordant (i.e., a long APD was associated with a large calcium transient) and regions that are electromechanically discordant (i.e., a long APD is associated with a small calcium transient).

### 3.2. Atrial Tissue Models

Atrial fibrosis is associated with pathological conditions such as persistent and permanent atrial fibrillation (AF). Ashihara et al. [42] hypothesized that electronic interactions between atrial myocytes and fibroblasts may play a role in the genesis of complex fractionated atrial electrograms (CFAE) and proposed that targeting these fibroblast-associated CFAE sites could terminate induced AF. Using a 2D sheet computational model of human atrial tissue with cardiomyocytes represented by the Courtemanche model [43] and the MacCannell fibroblast model, they showed that the incorporation of high-density fibroblasts (50% fibrotic area) resulted in more spiral wave meandering within or around the fibrotic areas and these meandering waves were sustained longer than controls without fibrotic areas. Moreover, computed bipolar electrogram recordings from the fibrotic areas showed CFAEs. Simulations of CFAE-targeted ablation resulted in spiral wave reentry termination shortly after ablation. This suggests that the fibroblast-mediated decrease in APD, CV, and myocardial excitability is required for CFAEs. In contrast, simulated collagen accumulation (i.e., low density (18.8%) or high density (37.5%) replacement of fibrotic area by nonexcitable and nonconductive tissue) showed less frequent wave breakups and no CFAE sites in the bipolar electrogram. However, the manifestation of such CFAEs could be related to the direction of the incident waveform relative to the underlying spatial organization of the fibrosis as demonstrated in a 2D computational model study by Campos et al. [44]. Catheter ablation targeting these collagen accumulation regions could not terminate spiral waves.

*Realistic 3D Computational Models*. McDowell et al. [45] developed a three-dimensional computational model of human left atrial tissue with specific geometry from a patient with persistent AF including models with combinations of GJ remodeling, collagen deposition, and myofibroblast proliferation with electronic or paracrine effects. They found that GJ remodeling was the primary contributor to conduction block and inclusion of other fibrotic lesions did not suppress it. Furthermore, all simulations which incorporated both GJ remodeling and myofibroblast coupling resulted in reentry after conduction block, thus indicating myofibroblasts are critical for reentry formation.

## 4. Mathematical Models of Gap Junctions

Given the arrhythmogenic implications of F-M coupling discussed above, a thorough understanding of F-M coupling could provide insights into the mechanisms of electrical dysfunction during cardiac fibrosis. Previous, computational studies exploring F-M coupling used a simplified “static” model of gap junction coupling, in which the conductance is represented as a constant value resistor. However, GJ channels are a diverse population of channels that vary in conductance and gating properties.

GJ channels are unique members of the ion channel family in that they span two lipid bilayer membranes. A single GJ channel is composed of two hemichannels (hC) docked head-to-head. Each hC is composed of six protein subunits, termed connexins (Cx), which are arranged in a hexagonal pattern around a central pore. This pore allows for direct communication between two neighboring cells. The extent of the electrical and metabolic transfer depends on the connexin isoform composition of the GJ channel. The adult heart predominantly expresses four Cx isoforms: mCx30.2, Cx40, Cx45, and Cx43. Each isoform exhibits unique static properties (i.e., channel number and conductance) and dynamic properties (voltage-sensitive gating and inactivation kinetics). These general time- and voltage-dependent behaviors of GJ channels have been incorporated into several computational models discussed below. In general, GJ channels are described as homotypic if both hCs are composed of the same Cx isoform, or heterotypic if the Cxs of the two hCs differ. Moreover, a given hC can be homomeric (composed of a single Cx isoform) or heteromeric (composed of multiple Cx isoforms).

### 4.1. Transjunctional-Voltage-Dependent Models

#### 4.1.1. Four-State Models

Recent models have described transjunctional voltage (*V*_j_)-gating of GJ channels using a four-state model. This representation describes each hC as having one voltage gate and thus two gates in series control the gating of the GJ channel. Each voltage gate can exist in an open or closed residual state. This results in four possible states: (1) OO, in which both gates are open, (2) CO, in which the left gate is close and the right gate is open, (3) OC, in which the left gate is open and the right gate is closed, and (4) CC, in which both gates are closed. This four-state scheme for *V*_j_-gating was used to develop a general mathematical model by Vogel et al., a steady-state model by Chen-Izu et al., and a stochastic model by Paulaskas et al.

**Figure 1:**
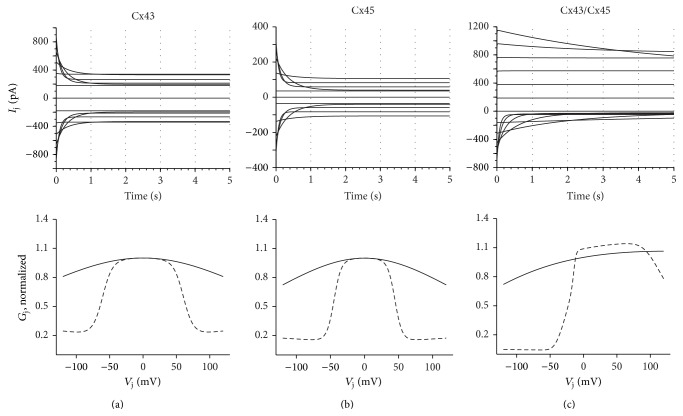
Simulations of homotypic Cx43, Cx45, and heterotypic Cx43/Cx45 GJ using the Vogel model. Parameters of the Vogel model were modified using least-squares curve fitting to experimental data. The models reproduce key features of the dynamic properties of GJ channels. The conductance (*G*_j_) of the homotypic Cx43 and Cx45 GJs is maximal at *V*_j_ = 0 and decreases symmetrically with increased *V*_j_. Comparison of the *G*_j_ versus *V*_j_ graphs of Cx43 (a) and Cx45 (b) shows that Cx45 has greater *V*_j_ sensitivity. This is in accordance with experimental data. Cx43/Cx45 GJs (c) show asymmetric voltage sensitivity, with enhanced *V*_j_ sensitivity at negative *V*_j_.

Vogel and Weingart [46] mathematical model is based on single channel data and consists of two hC models connected in series with each hC containing a voltage gate; each hC transitions between two nonzero states, a high (H) and low (L) conductance state gated by the transjunctional voltage, *V*_j_, across each hC. The gates function independently, leading to four conformation states of the combined channel, HH, HL, LH, and LL. This is different from ionic channels which typically have zero conductance at the closed state. Simulations of homotypic Cx45, Cx43, and heterotypic Cx43/Cx45 GJs using different formulations of the Vogel model are shown in Figure 1. Parameters were determined using least squares curve fitting of the model to experimental data extracted from Desplantez et al. [47]. The model reproduces key features of the dynamic properties of GJ channels. The gap junctional conductance (*G*_j_) of the homotypic Cx43 and Cx45 GJs is maximal at *V*_j_ = 0 and decreases symmetrically with increased *V*_j_. Comparison of the *G*_j_ versus *V*_j_ graphs of Cx43 Figure 1(a) and Cx45 Figure 1(b) shows that Cx45 has greater *V*_j_ sensitivity. This is in accordance with experimental data; the half-maximal inactivation voltage for Cx45 ranges from 23 to 30 mV, while that of Cx43 is 55–60 mV [48]. Cx43/Cx45 GJs Figure 1(c) show asymmetric voltage sensitivity, with enhanced *V*_j_ sensitivity at negative *V*_j_. This asymmetry facilitates current flow from the Cx45 expressing cell to the Cx43 expressing cell but impedes flow in the opposite direction, which may have important implications in arrhythmogenesis.

The Chen-Izu et al. [49] mathematical model focused on the steady state behavior of *V*_j_-gating. They developed a modified Boltzmann equation that allowed for the simultaneous fitting of positive and negative polarities of *V*_j_. The Chen-Izu model assumes that an intact gap junction channel has one open channel conductance and one residual conductance, as opposed to the Vogel model which has four possible conductances. Two models were developed, a contingent gating model in which the gating of one channel depends on the state of the opposed channel and an independent gating model which assumes that the two voltage gates in the model do not interact other than through the distribution of *V*_j_.

A four-state stochastic model of contingent gating of GJ channels containing two fast gates and *V*_j_ sensitive gating was developed by Paulaskas et al. [50]. The model assumes that each channel has an open state and a residual state and that both states rectify. Gates can have the same or different gating polarities. Each hC gate can be in the open or closed states which correspond to the open state and the residual state of the hC, respectively. The unitary conductances of the open and residual state rectify and thus depend on *V*_j_. The model defines for a given time whether individual channels stay in the same state or change their state. This model has also been extended into a stochastic 16-state model of voltage gating of GJ channels by incorporating the slow gating mechanism [51].

#### 4.1.2. Data-Based Model

Lin et al. [52] developed a model to describe the unique features of *V*_j_ gating between cardiomyocyte cells. This gating is thought to occur when CV is very slow (≤10 cm/s) and the intercellular conduction delay is large enough to produce *V*_j_ gradients equal to the magnitude of a ventricular AP. The major component of the model is the two inactivation components and two recovery components based directly from data obtained by applying a ventricular AP to paired neonatal murine ventricular myocytes in dual whole cell voltage clamp experiments. Inactivation is induced when *V*_j_ increases above a certain threshold value and is removed when *V*_j_ is in the resting state. They incorporated a behavior described as facilitation which occurs only in ventricular cardiomyocyte cell pairs. Facilitation is observed as an increase in *G*_j_ above the initial peak values during the final repolarization phase of the AP.

## 5. Simulations of Dynamic GJ Coupling

### 5.1. Effects on Myocyte-Myocyte Coupling

Henriquez et al. [53] used the Vogel model of dynamic GJ channels to couple 300 cells in a linear strand using the LR1 model. As stated above, the Vogel model represents the voltage- and time-dependent conductance of the GJ channels; the effects of this model were compared to a static GJ model with a constant value conductance. The results showed that when cells were tightly coupled (6700 GJ channels) little change was observed in the gap junctional conductance during propagation. However, for poor coupling (85 GJ channels), the gap junction conductance inactivates during propagation. This transient change in conductance resulted in increased transjunctional conduction delays, slowing of AP upstroke, and conduction block.

Lin et al. [54] simulated ventricular AP propagation using a 100-cell 1D cable model using the Faber and Rudy model of ventricular AP [55] and dynamic GJ coupling was modeled using their dynamic model for ventricular junctional conductance [52]. During normal conduction of 64 cm/s and *G*_j_ of 2500 nS there is very little change in CV by the introduction of the *V*_j_-dependent gating. However, the model predicted changes to be seen only when CV was below 10 cm/s when the *V*_j_ would mimic an AP. In this scenario, differences in CV between the static and the dynamic model were observed. For example, compared with the static model, the dynamic GJ model reduces CV by approximately a third (i.e., from 1.0 cm/s to <0.8 cm/s) at 6 nS of *G*_j_. Furthermore, modeling the effects of 100 nM dose of rotigaptide, a gap junctional conductance enhancer, resulted in a 60% prevention of the conduction slowing, thus preventing the formation of unidirectional block.

Casaleggio et al. [56] incorporated the rectification behavior often seen in heterotypic GJ channels by modeling a small 2D tissue sheet using the Beeler-Reuter model. They investigated the hypothesis that ischemia alters the properties of GJs inside the ischemic area by reducing the average gap junctional conductance, incorporating random fluctuations with time and by modifying the GJ rectifying properties along the edges of the ischemic area. These alterations alone resulted in the development of the main types of nonfatal arrhythmia behavior observed in experimental ECG recordings: single premature ventricular beats, trigeminy complexes, bigeminy complexes, couplets, triplets, and short runs of tachyarrhythmias. In the case of single premature ventricular beats, the main mechanism of arrhythmia formation is that in the presence of a lesion the signal propagation around the scar generates a secondary wave inside the ischemic region, once this wave reaches the normal region it causes a premature beat which then propagates backwards. Further simulations using different values for the average and variance of the gap junctional conductance inside the ischemic area found that for an average gap junctional conductance of 4.7 nS a higher variance results in a shift from isolated premature beats to other forms of arrhythmias. These results suggest that random fluctuations in the gap conductance inside an ischemic area can promote and modulate the development of specific types of arrhythmic behavior.

In summary, *V*_j_-dependent inactivation of GJs can alter myocyte-myocyte interactions and modify cardiac arrhythmia behavior under pathological conditions of decreased coupling as found in ischemic regions and border zones. However, to the best of our knowledge no previous attempts have been made to characterize the contribution of the dynamic GJ channel properties on F-M interactions. In the following section, we present investigations from our group into the potential role of *V*_j_-dependent gap junctions in F-M interactions.

### 5.2. Effects on Fibroblast-Myocyte Coupling

To provide mechanistic insight into which parameters play a key role in modifying the cardiomyocyte APD and morphology during GJ mediated F-M coupling, we performed simulations using a modified version of the Livshitz and Rudy guinea pig cardiomyocyte model [57] and coupled it to the MacCannell fibroblast model. The Vogel et al. mathematical model was chosen to represent the dynamic properties of the GJ channels because it can be modified to represent heterotypic GJs and it reproduces the key features of *V*_j_-gating of GJs (Figure 1):
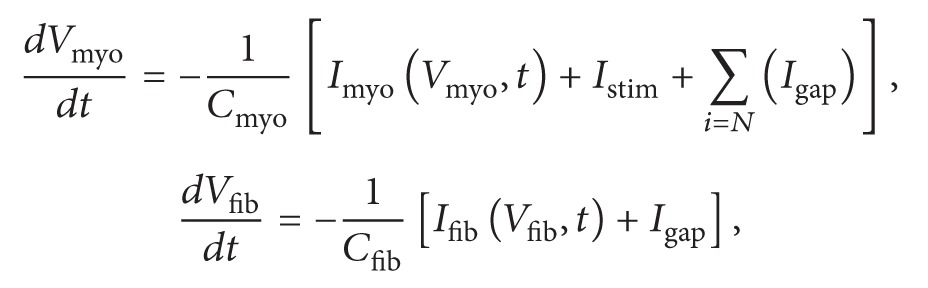
(1)

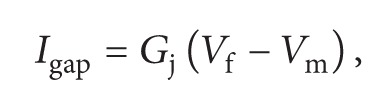
(2)

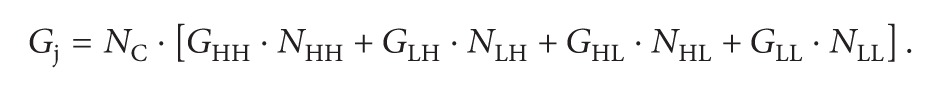
(3)To simulate F-M coupling, the myocyte (*V*_myo_) and the fibroblast (*V*_fib_) membrane voltage are derived from (1). *C*_myo_ and *C*_fib_ are the membrane capacitance and *I*_myo_ and *I*_fib_ are the total ionic current flowing through the ion channels, pumps, and exchangers. *N* is the number of fibroblasts coupled to the cardiomyocyte. The gap junctional current, *I*_gap_, is calculated using (2), where the gap junctional conductance, *G*_j_, is dependent on the model used (“static” or “dynamic”). In the static model, *G*_j_ is a constant value equal to the maximum value of the dynamic model. In the dynamic model the conductance is dependent on the fraction of GJs in a given state and their corresponding conductance where *N*_C_ is the number of GJ channels between the F-M pair (3).

**Figure 2:**
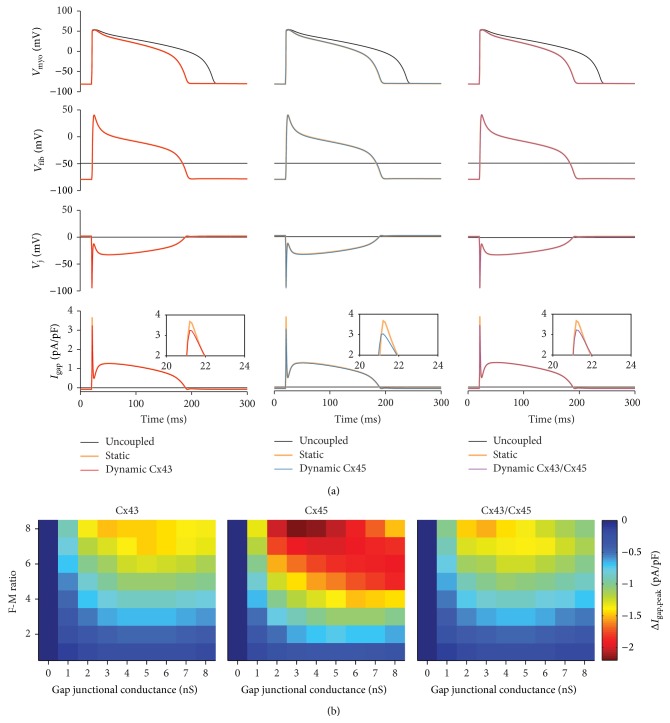
Simulations of F-M coupling pairs comparing coupling via dynamic Cx43, Cx45, and Cx43/Cx45 GJs to a static model. (a) Dynamic GJ models were used to couple the MacCannell fibroblast model to the modified Livshitz and Rudy model [57] and compared it to the static model and an uncoupled cardiomyocyte model. From top to bottom: membrane voltage of cardiomyocyte model *V*_myo_, membrane voltage of the fibroblast model (*V*_fib_), transjunctional voltage between the cardiomyocyte and fibroblast models (*V*_j_), and gap junction current injected into the cardiomyocyte (*I*_gap_). Uncoupled: control condition [black trace], no fibroblast coupled to cardiomyocyte. Static: constant value conductance [orange trace]. Dynamic: representing the Cx43 [left: red trace], Cx45 [center: blue trace], or Cx43/Cx45 [right: purple trace] dynamic GJ model. Parameters of the models were altered to represent a maximal 3 nS conductance and coupling to one fibroblast model. There is significant overlap between the static model and the dynamic model results. (b) The dependence of the gap junctional current on the gap junction phenotype is illustrated using pseudocolor plots of the difference in the peak gap junctional current (Δ*I*_gap,peak_) between the dynamic (Cx43, Cx45, or Cx43/Cx45) and the static model as a function of the F-M ratio and the gap junctional conductance.

Figure 2(a) demonstrates that F-M coupling including *V*_j_-gating of GJs results in a decrease in the AP plateau and APD as discussed in (Section 3.1.1). In addition, dynamic GJ gating during F-M coupling results in a novel mechanism to modulate the magnitude of the gap junctional current. Compared to static coupling, dynamic coupling and thus the dynamic properties of GJs reduce the early component of the gap junctional current. This is due to the inactivation of *G*_j_ at large negative *V*_j_ during the AP upstroke. The Cx45 GJ phenotype results in a larger reduction in the early component of gap junctional current compared to the Cx43 GJ phenotype.

In order to determine for which levels of fibrosis and magnitude of gap junctional conductance would *V*_j_-gating of GJs play a significant role, we modeled a single cardiomyocyte coupled to a varying number of fibroblasts representing normal (F-M ratio ≤2) to diseased levels of fibrosis (F-M ratio >2) [58]. In addition, we varied the magnitude of the gap junctional conductance across the range of experimentally measured values in cultured cells [59]. For each F-M coupling simulation, we then calculated the magnitude of the first peak of the gap junctional current when coupling was mediated by the three dynamic GJ models and when coupling was mediated by a static model. Δ*I*_gap,peak_ is the difference between the magnitudes of the peaks during dynamic GJ coupling and static coupling.  Figure 2(b) shows Δ*I*_gap,peak_ as a function of F-M ratio and gap junctional conductance. The largest changes in the difference current occur at high levels of fibrosis and intermediate values of gap junctional conductance (2–4 nS).

Therefore, dynamic GJ coupling can modify the relative magnitude of the early *I*_to_-like component of the gap junctional current as discussed in Section 3.1.1. Thus, dynamic GJ coupling may modify susceptibility to EADs and slow-rate alternans, which has been shown to be dependent on the early *I*_to_-like component of the gap junctional current as discussed in Section 3.1.4.

## 6. Conclusions

In conclusion, multiscale computational studies in combination with* in vitro* experiments have demonstrated that cardiac fibroblasts can modify action potential duration, induce spontaneous activity, modify conduction velocity, and increase susceptibility to early afterdepolarizations and cardiac alternans. The extent of these fibroblast-mediated changes depends on the density of fibrosis, the magnitude of the gap junctional conductance, and the underlying electrophysiology of cardiac fibroblasts.

We developed mathematical models of dynamic gap junctional channels that reproduce key features of the time- and voltage-dependent properties of Cx43, Cx45, and Cx43/Cx45 gap junctional channels and compared simulations of static and dynamic fibroblast-myocyte coupling. We showed that the early component of the gap junctional current was reduced during dynamic fibroblast-myocyte coupling and the magnitude of this reduction depends on the phenotype of the gap junctional channel, the magnitude of the gap junctional conductance, and the fibroblast-myocyte ratio.

However, many questions remain and require further investigation. First, more detailed experimental data and more refined computational models of cardiac fibroblasts and myofibroblast are needed to further characterize fibroblast-myocyte electronic interactions. Second, additional organ level computational studies of atrial and ventricular tissue will provide insight into how cellular and tissue level changes during fibroblast-myocyte coupling translates into organ level arrhythmogenesis. Finally, whether fibroblast-myocyte coupling occurs in the normal or diseased heart* in vivo* remains to be determined and is important to the understanding of the implications of fibroblast-myocyte interactions in the whole heart.
